# NSUN3-mediated mitochondrial tRNA 5-formylcytidine modification is essential for embryonic development and respiratory complexes in mice

**DOI:** 10.1038/s42003-023-04680-x

**Published:** 2023-03-22

**Authors:** Yoshitaka Murakami, Fan-Yan Wei, Yoshimi Kawamura, Haruki Horiguchi, Tsuyoshi Kadomatsu, Keishi Miyata, Kyoko Miura, Yuichi Oike, Yukio Ando, Mitsuharu Ueda, Kazuhito Tomizawa, Takeshi Chujo

**Affiliations:** 1 Department of Molecular Physiology, Faculty of Life Sciences, Kumamoto University, Kumamoto, 860-8556 Japan; 2 Department of Neurology, Faculty of Life Sciences, Kumamoto University, Kumamoto, 860-8556 Japan; 3 Department of Modomics Biology and Medicine, Institute of Development, Aging and Cancer, Tohoku University, Sendai, 980-8575 Japan; 4 Department of Aging and Longevity Research, Faculty of Life Sciences, Kumamoto University, Kumamoto, 860-0811 Japan; 5 Department of Molecular Genetics, Faculty of Life Sciences, Kumamoto University, Kumamoto, 860-8556 Japan; 6 Center for Metabolic Regulation of Healthy Aging, Kumamoto University, Kumamoto, 860-8556 Japan; 7 Department of Amyloidosis Research, Faculty of Pharmaceutical Sciences, Nagasaki International University, Sasebo, 859-3298 Japan

**Keywords:** RNA, Organelles

## Abstract

In mammalian mitochondria, translation of the AUA codon is supported by 5-formylcytidine (f^5^C) modification in the mitochondrial methionine tRNA anticodon. The 5-formylation is initiated by NSUN3 methylase. Human *NSUN3* mutations are associated with mitochondrial diseases. Here we show that *Nsun3* is essential for embryonic development in mice with whole-body *Nsun3* knockout embryos dying between E10.5 and E12.5. To determine the functions of NSUN3 in adult tissue, we generated heart-specific *Nsun3* knockout (*Nsun3*^HKO^) mice. *Nsun3*^HKO^ heart mitochondria were enlarged and contained fragmented cristae. *Nsun3*^HKO^ resulted in enhanced heart contraction and age-associated mild heart enlargement. In the *Nsun3*^HKO^ hearts, mitochondrial mRNAs that encode respiratory complex subunits were not down regulated, but the enzymatic activities of the respiratory complexes decreased, especially in older mice. Our study emphasizes that mitochondrial tRNA anticodon modification is essential for mammalian embryonic development and shows that tissue-specific loss of a single mitochondrial tRNA modification can induce tissue aberration that worsens in later adulthood.

## Abstract (web summary)

A mitochondrial tRNA modification enzyme, NSUN3, is found to be essential for embryonic development in mice, with whole-body knockout causing embryonic lethality and heart-specific Nsun3 knockout leading to heart aberration in older mice.

## Introduction

tRNA molecules function as adapters that convert genetic information transcribed in the form of mRNA into proteins^1,2^. tRNAs contain a variety of modified nucleosides that are post-transcriptionally incorporated by specific enzymes. These tRNA modifications play pivotal roles in maintaining tRNA structural integrity, biochemical stability, and codon-anticodon interactions^3,4^. The physiological importance of tRNA modifications is shown by the presence of more than 50 human tRNA modification enzymes whose mutations or expressional aberrations are associated with diseases that frequently manifest as brain dysfunction, cancer, diabetes, or mitochondrial diseases^3–5^.

In humans, protein synthesis takes place not only in the cytoplasm, but also within mitochondria, where 13 respiratory complex proteins are synthesized by translation of mRNAs using 22 tRNAs and two ribosomal RNAs (rRNAs) transcribed from mitochondrial DNA (mtDNA)^6^. The 22 human mt-tRNAs contain 18 kinds of modifications at 137 positions^7^, many of which are important for health. Mitochondrial disease collectively refers to a group of diseases caused by mitochondrial dysfunction. Mitochondrial disease-associated mutations have been reported in several nucleus-encoded mt-tRNA modification enzyme genes^8–14^, suggesting that mt-tRNA modifications play pivotal roles in intra-mitochondrial protein synthesis.

Moreover, whole-body knockouts (KO) of the mt-tRNA modification enzyme genes *Mto1* or *Mtu1* are embryonic lethal in mice^15,16^. *Mto1* encodes an mt-tRNA modification enzyme required for the synthesis of 5-taurinomethyluridine (τm^5^U) at the anticodon first nucleotide in five mt-tRNAs (mt-tRNA^Leu1^, mt-tRNA^Trp^, mt-tRNA^Gln^, mt-tRNA^Lys^, and mt-tRNA^Glu^). *Mtu1* encodes an mt-tRNA modification enzyme that introduces thiolation to three τm^5^U-containing mt-tRNAs, resulting in τm^5^s^2^U modification at the anticodon first nucleotide of three mt-tRNAs (mt-tRNA^Gln^, mt-tRNA^Lys^, and mt-tRNA^Glu^). In contrast to embryonic lethality in mice lacking *Mto1* or *Mtu1*, which encode enzymes that target the first nucleotide of mt-tRNA anticodon, reported mice lacking enzymes that target other regions of mt-tRNAs are viable. For example, mice lacking *Cdk5rap1*, which encodes an enzyme that methyl-thiolates the nucleotide adjacent to the mt-tRNA anticodon are viable^17^. Additionally, mice lacking *NOL1/NOP2/Sun domain family member 2* (*Nsun2*), which encodes a methyltransferase that targets the variable loop of mitochondrial and cytoplasmic tRNAs^18,19^, are viable and do not display an apparent mitochondria-related phenotype^18,20^.

**Fig. 1:**
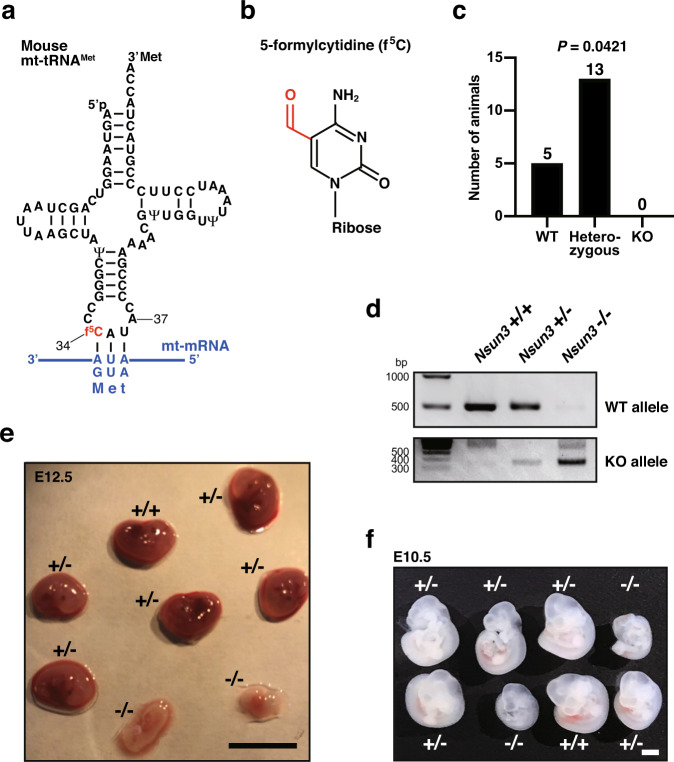
Embryonic lethality of whole-body *Nsun3* KO mice. **a** Secondary structure of the mouse mitochondrial (mt-) tRNA^Met^ with modified nucleosides: pseudouridine (Ψ) and 5-formylcytidine (f^5^C). The modifications are depicted based on human and bovine mt-tRNA^Met^ modifications^7,50^. The nucleoside position is numbered following conventional guidelines^51^. Note that f^5^CAU anticodon can base pair with two mitochondrial methionine-encoding mRNA codons AUG and AUA. **b** Chemical structure of f^5^C. The formyl modification at the cytidine *C5* position is shown in red. **c** Numbers of animals obtained by crossing parental heterozygous (*Nsun3*+/−) mice. *P* value was calculated by the chi-square test. **d** Genotyping analysis of embryos at stage E12.5. **e**, **f** Morphology of WT (+/+), heterozygous (+/−), and KO (−/−) embryos at stages E12.5 (**e**) and E10.5 (**f**) removed from the uterus of a heterozygous mother mouse. Scale bars, 5 mm (**e**) and 1 mm (**f**).

The human mitochondrial genetic code deviates from the canonical cytoplasmic genetic code. For example, the AUA codon, which encodes isoleucine in cytoplasmic translation, encodes methionine in mitochondria. To decode AUA as methionine, mt-tRNA^Met^ contains a 5-formylcytidine (f^5^C) modification in the anticodon first nucleotide^21^ (Fig. 1a, b). f^5^C enables the mt-tRNA^Met^ anticodon (CAU) to base pair with not only the AUG codon but also with the AUA codon^22^. f^5^C enables f^5^C-A pairing via imino-oxo tautomerization of the cytosine base, which is stabilized by the 5-formyl group^23^. f^5^C is synthesized by two mitochondrial matrix-localized enzymes, NSUN3 and AlkB homolog 1 (ALKBH1). After mt-tRNA^Met^ is transcribed, NSUN3 first methylates cytidine to form 5-methylcytidine, and ALKBH1 then oxidizes the methyl group to form a formyl group^24–27^. Due to the importance of f^5^C in mitochondrial translation, knockout of *NSUN3* or *ALKBH1* in cultured human cells, as well as mutation of *Nsun3* in mouse embryonic stem cells, result in a strong reduction of mitochondrial protein synthesis^25,26,28^.

Mitochondrial disease-associated mutations have been found in several nucleus-encoded mt-tRNA modification enzyme genes, such as *MTO1*, *GTPBP3*, *MTU1*, *TRMT10C*, *PUS1*, and *TRMT5*^8–12,14^. The mutations result in dysfunctions and developmental disorders in highly energy-consuming organs, including the heart, skeletal muscle, liver, and brain. Similar to the cases of other important mt-tRNA modification enzymes, mutations in the *NSUN3* gene are associated with mitochondrial diseases. One mitochondrial disease patient, who had compound heterozygous *NSUN3* mutations, developed symptoms of the disease at the age of 3 months, including muscle weakness, ophthalmoplegia, convergence nystagmus, increased plasma lactate level, microcephaly, and developmental delay^13^. Another mitochondrial disease patient with different compound heterozygous *NSUN3* mutations presented at the age of four months with muscle weakness, hypotonia, lactic acidosis, global developmental delay, and seizures^29^. In addition, a hypertension patient harboring a point mutation in the *mt-tRNA*^*Met*^ (A4435G in mtDNA) had thickening of his heart’s left ventricle posterior wall during his 60s and 70s^30^. This mutation corresponds to the 3′ adjacent nucleotide to the anticodon of mt-tRNA^Met^ (position 37 in the conventional tRNA position numbering) and has been found to decrease the efficiency of NSUN3-mediated mt-tRNA^Met^ modification in vitro^26^.

To investigate the physiological functions of NSUN3-mediated f^5^C modification, we generated *Nsun3* KO mice. Whole-body *Nsun3* KO mice were embryonic lethal, highlighting the importance of NSUN3 along with MTO1 and MTU1 as essential mt-tRNA anticodon modification enzymes for mouse embryonic development. These results establish that mt-tRNA anticodon modifications are crucial for mammalian embryonic development. Moreover, we showed that heart-specific *Nsun3* KO resulted in impaired heart respiratory complex activities and mild heart aberration, especially at an older age, indicating that tissue-specific loss of a single tRNA modification species in a single mt-tRNA can cause tissue aberration, especially in later adulthood.

## Results

### *Nsun3* is essential for embryonic development in mice

To investigate the physiological importance of NSUN3, we first attempted to generate whole-body *Nsun3* KO mice by crossing transgenic mice having exon 4 of the *Nsun3* gene floxed by LoxP sequence (*Nsun3*^*Flox/Flox*^) with transgenic mice carrying Cre recombinase under the control of cytomegalovirus enhancer and chicken *β-actin* (CAG) promoter. This resulted in the permanent deletion of targeted exons in the germ cells. The resulting *Nsun3*^*(Flox/*−*);CAGcre*^ mice were further crossed to C57BL/6 J mice to yield *Nsun3* heterozygous mice (*Nsun3*^+/−^). By mating *Nsun3*^+/−^ mice, we obtained five wild-type mice and 13 heterozygous mice, with no homozygous *Nsun3* KO mice obtained after multiple generations of breeding (Fig. 1c). We examined the morphology of embryos at embryonic day (E) 12.5 (Fig. 1d, e and Supplementary Fig. 1a). While the morphology of *Nsun3* heterozygous embryos did not differ from wild-type embryos, *Nsun3* KO embryos were small and appeared to start to become absorbed into mother’s uterus. At E10.5, while *Nsun3* KO embryos were smaller in comparison to wild-type or heterozygous embryos (Fig. 1f), heartbeats were observed in all *Nsun3* KO embryos. Thus, *Nsun3* KO embryos are alive at E10.5 but die before E12.5. These results clearly indicate that constitutive *Nsun3* deficiency leads to embryonic lethality in mice.

### Phenotypes in heart-specific *Nsun3* knockout mice

To clarify the possible roles of NSUN3-mediated tRNA f^5^C modification in adult tissue, we generated heart-specific *Nsun3* knockout (*Nsun3*^HKO^) mice. We chose to ablate *Nsun3* in the heart because the heart and skeletal muscle are the most susceptible tissues to mitochondrial dysfunction^31^. Another reason for choosing heart is that a hypertension patient having a *mt-tRNA*^*Met*^ mutation that can reduce NSUN3-mediated modification of mt-tRNA^Met^, showed left ventricle posterior wall thickening during his 60s and 70s^26,30^.

**Fig. 2:**
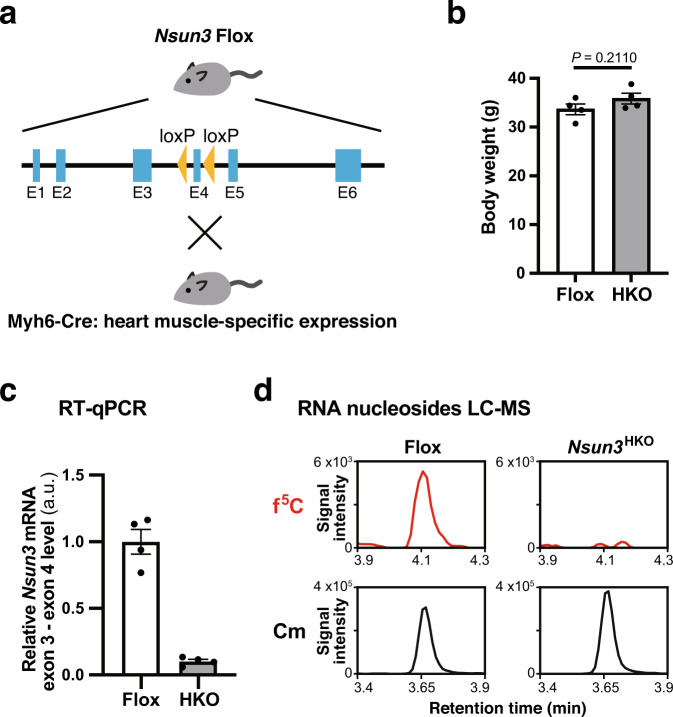
Generation of heart-specific *Nsun3* knockout (*Nsun3*^HKO^) mice. **a** Schematic illustration of the strategy to generate *Nsun3*^HKO^ mice. **b** Body weight of Flox mice and *Nsun3*^HKO^ mice at the time of sacrifice (13–20 weeks). Means ± s.e.m. from *n* = 4 mice. n.s. not significant by Welch’s *t*-test. **c** RT-qPCR of *Nsun3* mRNA exon 3–exon 4 using heart total RNA of 50-week-old Flox mice and *Nsun3*^HKO^ mice. The values were normalized by *Actb* mRNA levels. a.u. arbitrary units. Means ± s.e.m. from *n* = 4 mice. **d** LC-MS analysis of total RNA nucleosides made by nuclease P1 digestion of total RNA from mouse heart. Mass chromatograms detecting multiple reaction monitoring of f^5^C (Q1/Q3 = 272.20/140.20) or 2′-*O*-methylcytidine (Cm, a loading control, Q1/Q3 = 258.25/112.05) are shown. Q1/Q3: the mass of the single-protonated precursor ion and product ion.

*Nsun3*^HKO^ mice were generated by crossing transgenic mice harboring exon four of the *Nsun3* gene floxed by LoxP sequences (*Nsun3* Flox mice) with transgenic mice expressing Cre recombinase under the control of heart-specific Myosin heavy chain promoter (Myh6-Cre mice) (Fig. 2a). The *Nsun3*^HKO^ mice grew up without any obvious morphological defects, and adult *Nsun3*^HKO^ mice had equivalent body weights compared to the Flox mice (Fig. 2b). Heart muscle cell-specific *Cre* expression from the *Myh6* promoter resulted in the removal of most of *Nsun3* gene exon 4 in the heart, as confirmed by reverse-transcription quantitative PCR (RT-qPCR) (Fig. 2c). A small fraction of the remaining exon 4 in *Nsun3*^HKO^ heart may derive from non-heart muscle cells (e.g., blood vessel cells). Mass spectrometry analysis of heart total RNA nucleosides confirmed that f^5^C was absent in *Nsun3*^HKO^ hearts (Fig. 2d).

**Fig. 3:**
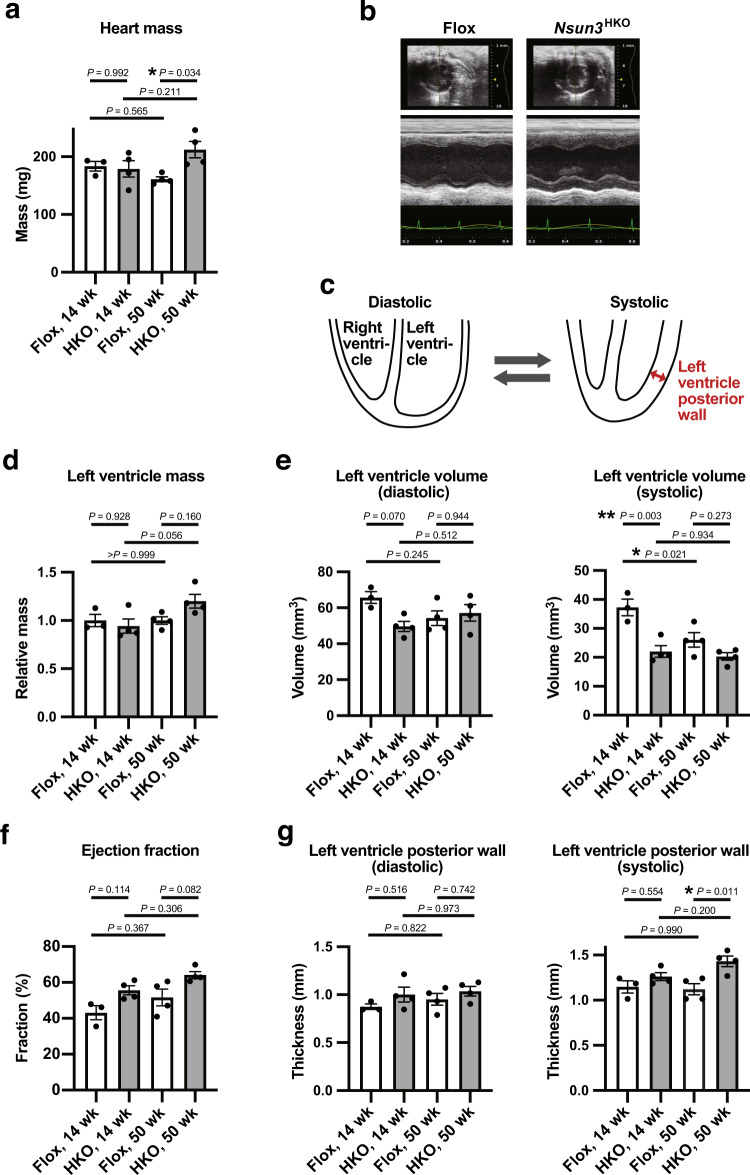
Heart aberrations in *Nsun3*^HKO^ mice. **a** The mass of 14- and 50-week-old mice hearts that were dissected and measured after echocardiography. **b** Representative M-mode echocardiography images of 50-week-old Flox mice and *Nsun3*^HKO^ mice. The upper images show the axis view of the left ventricle. Lower panels show the M-mode tracing of the left ventricle. **c** Schematic of diastolic stage and systolic stage of heart. **d** Left ventricle relative mass estimated by the echocardiography image analysis. **e** Left ventricle volume at diastolic stage (left panel) and systolic stage (right panel). **f** Calculated ejection fraction (%) of the hearts. **g** Left ventricle posterior wall thickness at diastolic stage (left panel) and systolic stage (right panel). Means ± s.e.m. from *n* = 3 mice (14-week-old Flox) or 4 mice (14-week-old *Nsun3*^HKO^, 50-week-old Flox and *Nsun3*^HKO^ mice). **P < 0.01 and *P < 0.05 by two-way ANOVA followed by Tukey’s test.

To investigate the impact of *Nsun3* deficiency on the heart, we first measured the mass of dissected hearts in 14-week-old young adult mice and 50-week-old mice (Fig. 3a). Although the *Nsun3*^HKO^ hearts showed equivalent weight as the control Flox mice at 14 weeks of age, *Nsun3*^HKO^ hearts were 31% heavier than Flox mice hearts at 50 weeks of age. Thus, at an older age, *Nsun3*^HKO^ hearts show mild enlargement, which often occurs as a compensatory response to compromised heart function.

To monitor heart function, we performed cardiac ultrasonography (Fig. 3b, c). The relative masses of the left ventricles, estimated by ultrasonography, were normal in 14-week-old, young adult *Nsun3*^HKO^ mice, but showed a slightly larger tendency in 50-week-old *Nsun3*^HKO^ mice, although the difference was statistically insignificant (Fig. 3d). On the other hand, left ventricle volume decreased in the systolic phase of *Nsun3*^HKO^ hearts at 14 weeks (Fig. 3e). Accordingly, although statistically insignificant, the ejection fraction showed an increasing tendency in *Nsun3*^HKO^ mice hearts (Fig. 3f). In addition, the left ventricle thickness increased in the systolic phase of 50-week-old *Nsun3*^HKO^ heart (Fig. 3g), suggesting enhanced heart contraction. Collectively, our results demonstrate that heart *Nsun3* knockout causes the development of mild heart abnormalities that become more apparent at an older age.

### Aberrant mitochondrial morphology in *Nsun3*^HKO^ mouse heart

**Fig. 4:**
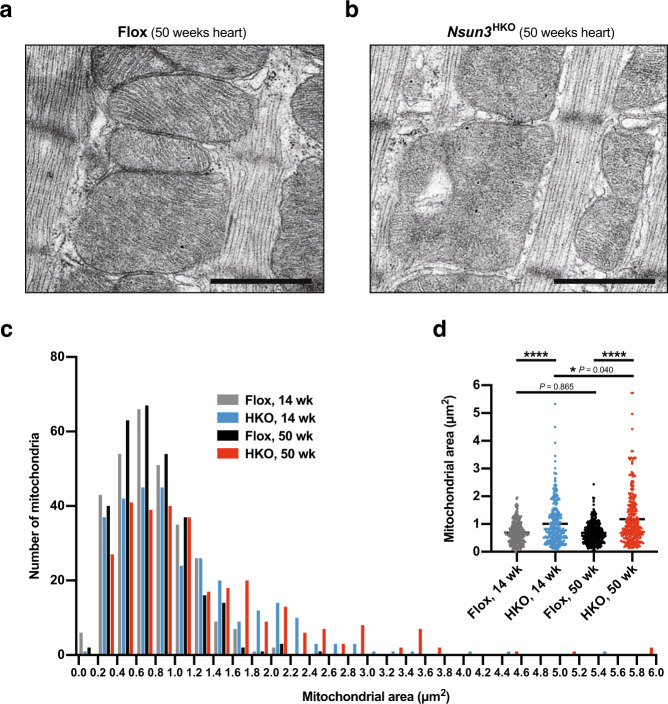
Morphological abnormalities of *Nsun3*^HKO^ mouse heart mitochondria. **a**, **b** Representative images of mitochondria in cardiac muscles of 50-week-old Flox mice (**a**) and *Nsun3*^HKO^ mice (**b**). Scale bar, 1 μm. **c** Histogram showing the size distribution of cardiac mitochondria from 14- or 50-week-old, Flox, or *Nsun3*^HKO^ mice. *n* = 300 mitochondria in each group were analyzed. **d** Violin plot of the same data as shown in the histogram. The mean mitochondrial areas (Flox 14-wk, 0.690 μm^2^; HKO 14-wk, 1.011 μm^2^; Flox 50-wk, 0.685 μm^2^; HKO 50-wk, 1174 μm^2^) are indicated by horizontal lines. ****P < 0.0001 and *P < 0.05 by Mann–Whitney test.

Abnormal mitochondrial morphology is a hallmark of mitochondrial dysfunction. Since NSUN3 is a mt-tRNA^Met^ modification enzyme required for efficient mitochondrial translation^13,24,26^, we next examined mitochondrial morphology using transmission electron microscopy. Mitochondria in the cardiac muscle of Flox control mice were filled with well-organized, elongated cristae structures (Fig. 4a). By contrast, the *Nsun3*^HKO^ heart mitochondria had fragmented cristae structures (Fig. 4b). Metabolic needs due to impairment of mitochondrial function can promote mitochondrial remodeling as a compensation mechanism^32,33^. Indeed, quantification of the mitochondrial size revealed that the mean size of *Nsun3*^HKO^ heart mitochondria (1.011 μm^2^) was 1.5 times larger than the mean size of Flox heart mitochondria (0.690 μm^2^) at 14 weeks of age and 1.7 times larger at 50 weeks of age (Flox: 0.685 μm^2^, *Nsun3*^HKO^: 1.174 μm^2^) (Fig. 4a–d). In addition, 50-week-old *Nsun3*^HKO^ heart mitochondria were 17% larger than 14-week-old *Nsun3*^HKO^ heart mitochondria (Fig. 4d). These aberrant mitochondrial morphologies indicated that *Nsun3*^HKO^ mice may have dysfunctional heart mitochondria.

### *Nsun3*^HKO^ does not decrease the steady-state levels of heart mitochondrial tRNAs and mRNAs

**Fig. 5:**
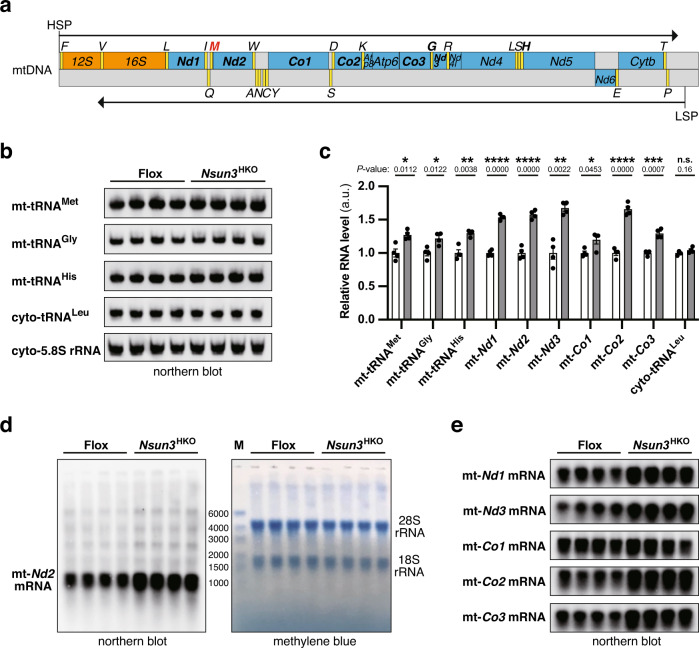
The steady-state levels of mt-tRNAs and mt-mRNAs in *Nsun3*^HKO^ mouse heart. **a** Schematic of the linearized mtDNA structure consisting of tRNA genes (yellow), protein-coding genes (blue), rRNA genes (orange), and noncoding regions (gray). Polycistronic precursor RNAs are transcribed from the heavy-strand promoter (HSP) and light-strand promoter (LSP), followed by cleavages at the 5′ and 3′ sides of tRNAs to produce respective RNAs. The *mt-tRNA*^*Met*^ gene is indicated in red, and genes encoding northern blotted RNAs are indicated in bold letters. **b** Northern blot analysis of heart tRNAs from 14-week-old, *n* = 4 Flox and *Nsun3*^HKO^ mice. Cytoplasmic 5.8 S rRNA is shown as a loading control, and cytoplasmic tRNA^Leu^_CAA_ is shown as a comparison to mt-tRNAs. **c** Quantification of tRNAs in (**b**) and mt-mRNAs in (**d**) and (**e**). tRNA was normalized by 5.8 S rRNA, and mRNA was normalized by 28 S rRNA. Means ± s.e.m. from *n* = 4 Flox and *Nsun3*^HKO^ mice. ****P < 0.0001, ***P < 0.001, **P < 0.01, and *P < 0.05 by Welch’s *t*-test. **d** Northern blot analysis of heart mt-*Nd2* mRNA from the same mice used in tRNA analysis. Mature mt-*Nd2* mRNA is 1038 nt plus poly(A) tail of up to 50 nt. The methylene blue-stained membrane used for the mt-*Nd2* mRNA northern blot is shown on the right to monitor RNA transfer. M indicates size marker. **e** Northern blot analysis of heart mt-mRNAs from the same mice as in the above analyses. All of the mtDNA-encoded complex IV subunit mRNAs (mt-*Co1*, mt-*Co2*, and mt-*Co3*) were monitored.

Mitochondrial RNAs are transcribed as polycistronic precursors and then processed into each RNA species^6,34^ (Fig. 5a), and the stability of mature mt-RNAs is post-transcriptionally regulated by RNA-binding proteins and RNases in mitochondria^35^. To evaluate the effects of *Nsun3* loss on mitochondrial RNA steady-state levels, we conducted northern blots of heart mt-tRNAs and mt-mRNAs. As a result, we observed a slight increase in the steady-state levels of all monitored mt-tRNAs and mt-mRNAs, including mt-tRNA^Met^ (Fig. 5b–e and Supplementary Fig. 2). This result indicates that mt-tRNA^Met^ steady-state level increased likely due to increased mitochondrial volume (Fig. 4) and/or mitochondria-wide RNA upregulation, rather than an event specific to mt-tRNA^Met^. The mt-*Nd2* mRNA is directly connected to mt-tRNA^Met^ within the polycistronic precursor (Fig. 5a). To assess whether the loss of f^5^C modification in mt-tRNA^Met^ affects processing at the mt-tRNA^Met^-*Nd2* boundary, the entire membrane of mt-*Nd2* northern blot is shown in Fig. 5d. We observed only some increase of the precursor RNA (faint bands observed above mature mt-*Nd2*) at a comparable level to the increase in mature mt-*Nd2* mRNA level, which suggests that the loss of f^5^C modification in mt-tRNA^Met^ has a minimal or no effect on mt-tRNA^Met^-*Nd2* boundary processing. Overall, these results indicate that *Nsun3*^HKO^ does not decrease the steady-state levels of observed mature mt-tRNAs and mt-mRNAs.

### *Nsun3*^HKO^ causes mitochondrial respiratory complex dysfunction exacerbated at an older age

**Fig. 6:**
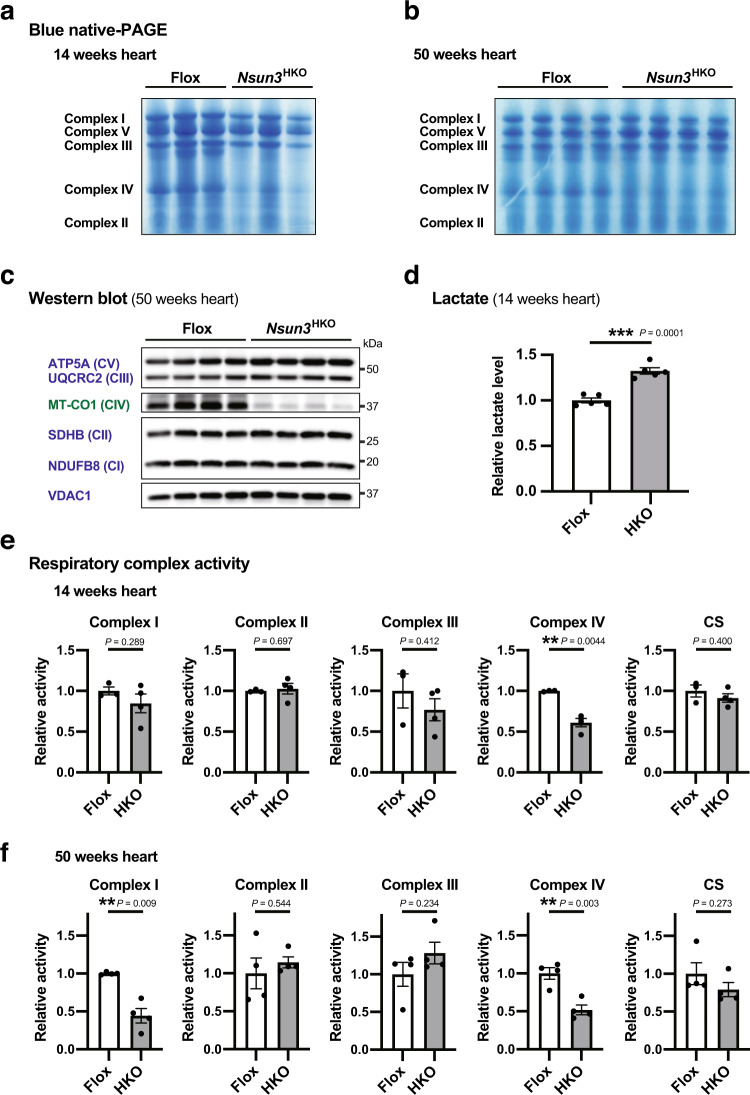
Dysfunction of specific respiratory complexes in *Nsun3*^HKO^ mouse heart. **a**, **b** Blue native-PAGE of respiratory complexes of 14-week-old (**a**) and 50-week-old (**b**) mouse heart mitochondria. **c** Western blot analysis of complexes I–V proteins in 50-week-old mice hearts. mtDNA-encoded MT-CO1 is shown in green and nuclear DNA-encoded proteins are in blue. VDAC1 is a loading control of mitochondrial lysate. **d** Relative lactate levels in the hearts of 14-week-old mice. Means ± s.e.m. from *n* = 5 mice each. ***P < 0.001 by Welch’s *t*-test. **e**, **f** Relative activities of respiratory complexes I-IV in 14-week-old (**e**) and 50-week-old (**f**) mice heart mitochondria. CS citrate synthase activity, measured as a loading control. Means ± s.e.m. from *n* = 3 mice (14-week-old Flox) or 4 mice (14-week-old *Nsun3*^HKO^, 50-week-old, Flox or *Nsun3*^HKO^ mice). **P < 0.01 by Welch’s *t*-test.

We next evaluated the quantity and activities of mitochondrial respiratory complexes in 14- and 50-week-old mice hearts. To quantify respiratory complexes, mitochondria were fractionated from 14- and 50-week-old mice hearts and whole respiratory complexes were detected by blue native-PAGE. In *Nsun3*^HKO^ heart mitochondria, we observed a decrease of complex IV in 14-week-old or 50-week-old heart mitochondria (Fig. 6a, b and Supplementary Fig. 1b). Accordingly, the steady-state level of MT-CO1 protein, a mtDNA-encoded complex IV protein, was markedly decreased in *Nsun3*^HKO^ mice (Fig. 6c and Supplementary Fig. 1c, d). By contrast, the steady-state levels of mt-mRNAs, including mRNAs of all of the mtDNA-encoded complex IV proteins (mt-*Co1*, mt-*Co2*, and mt-*Co3* mRNAs), were not decreased (Fig. 5c), consistent with the role of NSUN3-mediated tRNA^Met^ modification in the translation of mt-mRNAs rather than their stability. In the *Nsun3*^HKO^ hearts, we observed a mild increase in lactate levels (Fig. 6d), which may indicate that glycolysis activity was enhanced, possibly in response to decreased respiratory complex activity in the *Nsun3*^HKO^ hearts. Thus, finally, we measured the respiratory complex activities of 14- and 50-week-old heart mitochondria. The 14-week-old, young adult *Nsun3*^HKO^ heart mitochondria showed a decrease in complex IV activity (Fig. 6e). Moreover, 50-week-old *Nsun3*^HKO^ heart mitochondria showed an additional decrease in complex I activity compared to 14-week-old *Nsun3*^HKO^ (as seen by comparing Fig. 6e, f, *P* = 0.037, Welch’s *t*-test). Thus, *Nsun3*^HKO^ causes dysfunction of specific mitochondrial respiratory complexes, and the dysfunction exacerbates at an older age.

## Discussion

In this study, we first demonstrated that NSUN3, the enzyme required for f^5^C modification of the mammalian mt-tRNA^Met^ anticodon first nucleotide, is essential for embryonic development in mice (Fig. 1). The first nucleotide of tRNA anticodon is responsible for proper recognition of the mRNA codon third nucleotide, and loss of NSUN3-mediated f^5^C disables efficient decoding of AUA codons in mt-mRNAs^22^. Embryonic lethality of KO mice of other mt-tRNA anticodon modification enzymes, MTO1 (for τm^5^U modification) and MTU1 (for 2-thiolation in τm^5^s^2^U modification), emphasizes the pivotal roles of mt-tRNA anticodon first nucleotide modifications in mammalian embryonic development.

Our study demonstrates that the loss of *Nsun3* leads to abnormality in the heart and confirms the importance of *Nsun3* in mitochondrial function (Figs. 3, 4, 6). Heart-specific *Nsun3* KO resulted in decreased mitochondrial respiratory complex activities, fragmented mitochondrial cristae structures, and mitochondrial enlargement. Interestingly, although the *Nsun3*^HKO^ heart displayed some abnormalities in young adulthood (14 weeks of age), aberrant heart phenotypes were more apparent in later adulthood (50 weeks of age). This age-exacerbated phenotype is similar to a mitochondrial tRNA^Met^ mutant patient who was diagnosed with hypertension at the age of 44 and experienced thickening of left ventricle posterior wall at the age of 60s and 70s^30^. In a later report, this tRNA^Met^ mutation was shown to reduce the efficiency of NSUN3-mediated tRNA^Met^ modification in vitro^26^. Our work clearly indicates that deficiency of NSUN3-mediated f^5^C modification in the heart is associated with heart aberrations, especially at an older age.

Notably, the phenotypes of *Nsun3*^HKO^ are weaker compared to the heart-specific *Mto1* KO (*Mto1*^HKO^) mice that were previously reported^15^. *Mto1*^HKO^ mice were born, but did not survive longer than 24 h, whereas *Nsun3*^HKO^ mice grew up to adults. The enlargement of heart mitochondria in *Mto1*^HKO^ is more pronounced than in *Nsun3*^HKO^. *Mto1* knockout causes cytoplasmic unfolded protein responses, due to the accumulation of protein aggregates in the cytoplasm caused by impaired mitochondrial protein import from the cytoplasm^15^. By contrast, the *Nsun3*^HKO^ mouse heart did not show upregulation of unfolded protein response marker mRNAs *Xbp1* or *Chop* (Supplementary Fig. 3). Furthermore, while E9 embryos of whole-body *Mto1* KO or *Mtu1* KO are drastically smaller than wild-type and show aberrant morphologies^15,16^, whole-body *Nsun3* KO embryos at E9.5 exhibited relatively milder phenotype with moderately smaller body size than wild-type (as shown in Supplementary Fig. 4) and continued growing at least until E10.5 (Fig. 1f). The differences in the severity of phenotypes between *Nsun3*, *Mto1*, and *Mtu1* knockouts may be partially attributed to the numbers of mt-tRNAs that the corresponding tRNA modifications are introduced to. NSUN3 modifies only mt-tRNA^Met^, whereas MTO1 modifies five mt-tRNAs (mt-tRNA^Leu1^, mt-tRNA^Trp^, mt-tRNA^Gln^, mt-tRNA^Lys^, and mt-tRNA^Glu^) and MTU1 modifies three mt-tRNAs (mt-tRNA^Gln^, mt-tRNA^Lys^, and mt-tRNA^Glu^)^26,36^. Genetically inherited disorders caused by mt-tRNA modification deficiency are generally regarded to occur during embryonic development or at a young age^3,5^. The smaller number of NSUN3-modified tRNAs compared to MTO1-modified tRNAs may be the cause of relatively mild *Nsun3*^HKO^ heart aberrations, which became more apparent in late adulthood, in contrast to the strong disorders from young ages in *Mto1*^HKO^.

The reported human patients who have compound heterozygous mutations in the *NSUN3* gene were diagnosed to develop the mitochondrial disease at several months of age^13,29^. These infants presented with symptoms of mitochondrial diseases, such as lactic acidosis and skeletal muscle weakness, but heart failure was not reported. On the other hand, a mt-tRNA^Met^ mutation (A4435G mutation in mtDNA) was associated with hypertension and progressive thickening of the posterior wall of the left ventricle during his 60s and 70s, but was not associated with other clinical features^30^. This mutation site is located next to the mt-tRNA^Met^ anticodon (position 37 according to the conventional tRNA position numbering), and in vitro experiments have shown that it reduces the efficiency of NSUN3-mediated methylation to about 40%^26^. The patient with the mt-tRNA^Met^ mutation had a relatively mild phenotype compared to patients with *NSUN3* mutations, possibly due to the presence of some f^5^C in mt-tRNA^Met^. These previous studies and our results collectively suggest that patients with *NSUN3* mutations should be closely monitored for a potential decline in heart function as they age.

Upon *Nsun3*^HKO^, among the five respiratory complexes, the strongest phenotypes were seen in complexes IV and I; *Nsun3*^HKO^ resulted in a decreased complex IV steady-state level and decreased complex I and IV activities in older mice, and did not substantially affect other complexes (Fig. 6). One possible cause could be due to the number of AUA codons in mt-mRNAs; the numbers of mouse mt-mRNA AUA codons for each respiratory complex are 140 (complex I), 0 (complex II), 18 (complex III), 46 (complex IV), and 13 (complex V). In previous studies, similar to our *Nsun3*^HKO^ mice, knockout of mt-tRNA anticodon modification enzymes such as human *ALKBH1*, mouse *Mto1*, or mouse *Mtu1* all resulted in a marked decrease in activities of respiratory complexes I and/or IV, and lesser extent or no effects on complexes II and III^15,16,37^ (complex V activity cannot be measured by conventional methods). The biased effects of mt-tRNA anticodon modification enzyme knockouts to complexes I and IV may be due to the number of subunits that mtDNA encodes; mtDNA encodes seven subunits of complex I, no subunit of complex II, one subunit of complex III, three subunits of complex IV, and two subunits of complex V.

This study does not reveal the specific mechanisms between respiratory complex dysfunction and heart abnormalities. However, previous studies have shown various mechanisms by which respiratory complex dysfunctions can lead to progressive heart deficiencies^38^. For example, dysfunction of complex IV can halt the flow of electrons from NADH via complexes I and III, inducing leakage of electrons and production of reactive oxygen species (ROS)^39^. ROS overload can directly damage tissue and also open holes in the mitochondrial inner membrane, releasing cytochrome c and triggering cell death^40^. Furthermore, deficiency in oxidative phosphorylation can cause the heart to increase glycolysis for ATP generation, leading to elevated glucose uptake into the cells. A recent study suggests that high intracellular glucose uptake can lead to the accumulation of branched-chain amino acids via transcriptional rewiring, activating mTOR and causing cardiomyocyte hypertrophy^41^. It is possible that the reduced function of complex IV (Fig. 6e, f) and increased glycolysis in *Nsun3*^HKO^ hearts (as suggested by the increased heart lactate level in Fig. 6d) to slightly activate these pathways and result in some thickening of the left ventricular posterior wall.

Previous studies have shown that the f^5^C modification of mt-tRNA^Met^ mediated by NSUN3 plays a crucial role in maintaining the level of mitochondrial translation in human and mouse cells^13,24,26,28^. Translation levels are determined at both the initiation and elongation steps, and mt-tRNA^Met^ is used in both. An in vitro study has suggested a role for mt-tRNA^Met^ f^5^C modification in the initiation step of translation at the AUA codon and not the AUG codon^42^. Additionally, f^5^C modification was shown to enhance the efficiency of the elongation step of AUA codon translation, but had little effect on the AUG codon translation in vitro^22^. In *Nsun3*^HKO^ hearts, complex IV was the most affected respiratory complex (Fig. 6), although all of the mtDNA-encoded complex IV subunit mRNAs (mt-*Co1*, mt-*Co2*, and mt-*Co3*) use AUG as their initiation codons (Supplementary Table 1). Thus, in the *Nsun3*^HKO^ heart, the translation elongation step, rather than the initiation step, may be involved in the reduction of the complex IV level.

The role of f^5^C in the initiation of mitochondrial translation requires further studies. This is because, in addition to AUG and AUA, mammalian complex I and V mt-mRNAs also use AUU, AUC, and GUG codons as initiation codons (Supplementary Table 1). Additionally, the loss of *Nsun3* loss leads to a decrease in complex I activity in the *Nsun3*^HKO^ heart at an older age (Fig. 6f) and a decrease in the translation of mtDNA-encoded complex I, III, and V proteins in human and mouse cells^26,28^. The initiation step of mitochondrial translation is different from that of bacterial or cytoplasmic translation in various ways^43^. Therefore, to understand the potential role of mt-tRNA^Met^ f^5^C modification in translational initiation at AUU, AUC, and GUG codons, it would be necessary to conduct an in vitro translation experiment that uses mitochondrial ribosomes (rather than *E. coli* ribosomes) and other mitochondrial factors.

Regarding the physiological roles of NSUN3, a lack of understanding remains of the embryonic lethal phenotype of whole-body *Nsun3* KO and the relatively weak phenotype of *Nsun3*^HKO^. Although the heart is regarded as one of the most susceptible organs to mitochondrial dysfunction at postnatal stages^31^, the role of other tissues or cells for which mt-tRNA anticodon modifications play critical roles during the embryonic stage remains unclear. This question also arises with respect to the embryonic lethality of whole-body *Mto1* KO or *Mtu1* KO and viability of previously generated heart- or liver-specific *Mto1* or *Mtu1* KO mice^15,16^. Therefore, identifying the specific tissue(s) and stage(s) at which mt-tRNA modifications is critical during embryonic development will be a crucial question for mitochondrial biology and RNA biology.

## Methods

### Animals

Whole-body *Nsun3* knockout mice were generated by crossing transgenic mice having exon 4 of the *Nsun3* gene floxed by the LoxP sequence (*Nsun3*^*Flox/Flox*^) with transgenic mice carrying Cre recombinase under the control of cytomegalovirus enhancer and chicken *β-actin* (CAG) promoter. This crossing resulted in the permanent deletion of targeted exons in the germ cells. The resulting *Nsun3*^*(Flox/*−*);CAGcre*^ mice were further crossed to C57BL/6 J mice to yield *Nsun3* heterozygous mice (*Nsun3*^+/−^).

Heart-specific *Nsun3* knockout mice were generated by crossing transgenic mice in which the *Nsun3* gene exon 4 was floxed by LoxP sequences (*Nsun3* Flox mice), with transgenic mice expressing Cre recombinase under the control of *Myh6* promoter (Myh6-Cre mice). *Nsun3* Flox mice were backcrossed with C57BL/6 J mice for at least five generations to control for genetic background. Myh6-Cre mice were acquired previously^15^. Male mice were utilized for experiments, while female mice were primarily used for breeding purposes. Experiments were performed at 14 or 50 weeks of age. Mice were housed at 25 °C in a 12-h light and 12-h dark cycle. All animal procedures were approved by the Animal Ethics Committee of Kumamoto University (Approval ID: A2021-012R2).

### Genotyping

Genomic DNA was extracted from a 3–5 mm piece of tissue clipped from the end of the tail of 4-week-old mice. Approximately 50 ng of genomic DNA was subjected to PCR to detect the WT and KO alleles using KAPA 2 G Robust HotStart ReadyMix (KAPA Biosystems, Boston, USA), or floxed allele and Myh6-Cre alleles using KOD FX DNA polymerase (TOYOBO Life Science, Tokyo, Japan) following the manufacturer’s instructions. The primers are listed in Supplementary Table 2.

### Observation of embryos

Whole-body *Nsun3*^+/−^ males and females were paired overnight. The next morning, males were removed from the cages. The weight of females was checked on the day before observing embryos to estimate pregnancy. To observe E12.5, E10.5, or E9.5 embryos, the female mice were euthanized by isoflurane or cervical dislocation. The uterus was quickly opened and embryos were observed in phosphate-buffered saline (PBS) under a Stemi305 stereomicroscope (Zeiss, Oberkochen, Germany).

### RNA extraction

Mouse hearts were dissected and homogenized in 3 mL of TRI Reagent (MRC, Cincinnati, USA) using TissueRuptor (Qiagen, Hilden, Germany). The heart lysate in TRI Reagent was then centrifuged at 10,000 × *g* for 10 min, and the supernatant was used for total RNA extraction according to the manufacturer’s protocol.

### Reverse-transcription quantitative PCR (RT-qPCR)

RT-qPCR was performed as described previously^44^. cDNA was synthesized using 500 ng of total RNA and Prime-Script RT Master Mix (Takara, Kusatsu, Japan) according to the manufacturer’s protocol. Quantitative real-time PCR was then performed using the Rotor-Gene Q MDx 5plex HRM machine (Qiagen, Hilden, Germany) and TB Green Premix Ex Taq II (Takara) according to the manufacturer’s instructions. The primer sequences are listed in Supplementary Table 2.

### RNA nucleoside mass spectrometry

RNA nucleoside mass spectrometry was performed as previously described in refs. ^45–47^. A 25 μL solution containing 3 μg of heart total RNA, 20 mM Hepes-KOH (pH 7.6), 2 units of Nuclease P1 (Fujifilm, Tokyo, Japan), and 0.25 units of bacterial alkaline phosphatase (Takara, Kusatsu, Japan) was incubated at 37 °C for 3 h. About 3 μL of the nucleoside solution was then injected into the LC-MS-8050 system (Shimadzu, Kyoto, Japan). The nucleosides were first separated by an Inertsil ODS-3 column (GL Science, Tokyo, Japan) using a mobile phase that continuously changed from 100% of solution A (5 mM ammonium acetate in water, pH 5.3) to 100 % of solution B (60% acetonitrile in water) in 17 min at a flow rate of 0.4 mL min^−1^, followed by electrospray ionization and a triple quadrupole mass spectrometry in the multiple reaction monitoring modes.

### Echocardiography

Mice were preconditioned by chest hair removal using a topical depilatory (FujiFilm VisualSonics, Toronto, Canada), anesthetized with 1.5–2.5% isoflurane administered via inhalation, and maintained in a supine position on a platform with limbs attached for electrocardiogram gating during imaging. Body temperature was kept constant by feeding the signal of a rectal probe back to a heating pad, while heart and respiratory rates were continuously monitored. Transthoracic echocardiography was performed using a high-frequency ultrasound system for small animal imaging (VisualSonics Vevo 2100, FujiFilm VisualSonics, Toronto, Canada) using an MS 400 linear array transducer (18–38 MHz). M-mode recording was performed at the midventricular level. All images were analyzed using Vevo 2100 version 1.4 software. Left ventricle wall thickness and internal cavity diameters at diastole and systole were measured. Left ventricle volumes in diastolic phases (LV Vol d) and systolic phases (LV Vol s) were measured. The ejection fraction (%) was calculated as [(LV Vol d) - (LV Vol s)] (LV Vol d)^−1^ × 100. All procedures were performed under double-blind conditions with regard to genotype or treatment.

### Electron microscopy

Transmission electron microscopy examination was performed essentially as described previously in ref. ^48^. Briefly, heart tissues were first fixed in a solution containing 2% paraformaldehyde and 2% glutaraldehyde, cut in the fixative, and then additionally fixed at 4 °C for more than 2 h. The tissues were then washed, post-fixed in 1% OsO_4_ at 4 °C for 1 hour, washed and stained with 1.5% uranyl acetate at 4 °C for 1 h. After dehydration in ethanol and propylene oxide, the tissues were embedded in epoxy resin for 3 h and then polymerized at 60 °C for more than 48 h. The tissues were trimmed, cut into ~60 to 70 nm sections, and stained with 1.5% uranyl acetate for 10 min and with lead citrate for 10 min. Random sections were obtained from three hearts per group. Images were acquired at 80 kV on a HITACHI 7700 transmission electron microscope (Hitachi, Tokyo, Japan). The mitochondrial areas in images taken at 2500 × magnification were quantified using ImageJ software.

### Northern blot

For the tRNA northern blot, total heart RNA (1.5 µg) was separated using 7 M urea/TBE/10% PAGE at 150 V. The gel was then stained with SYBR Gold (Invitrogen, Carlsbad, USA) to assess the RNA quality and then transferred to a nylon membrane (Merck Millipore, Billerica, USA) using a wet transfer blotting system (Bio-Rad, Hercules, USA) on the ice at 50 V for 80 min. For mRNA northern blot, 1.8 µg of total heart RNA or 1.5 µg of RNA ladder (Nippon Gene, Tokyo, Japan) was separated using 6.7% formaldehyde/1xMOPS/1.2% agarose gel at 100 V. The RNA was then transferred to a nylon membrane (Merck Millipore, Billerica, USA) by an overnight, conventional sponging method using 20 × SSC. The next day, the membrane was briefly washed with MilliQ water, stained with methylene blue (MRC, Cincinnati, USA), and photographed. For both tRNA and mRNA northern blot, membranes were crosslinked with UV light at 1200 × 100 µJ cm^−2^ using HL-2000 Hybrilinker (Funakoshi, Tokyo, Japan) and incubated in prehybridization buffer (6 × SSC, 0.1% SDS, and 1 × Denhardt’s solution) at 42 °C for 1 h. The membranes were then hybridized with DIG-labeled (Roche, Basel, Switzerland) probe DNA in hybridization buffer (900 mM NaCl, 90 mM Tris-HCl pH 8, 6 mM EDTA, and 0.3% SDS) overnight at 50 °C. The membranes were washed with 1 × SSC, blocked using DIG wash and block buffer set (Roche), and probed with anti-DIG alkaline phosphatase Fab fragments (Roche) and CDP-Star (Roche). Images were taken by ImageQuant (GE Healthcare, Chicago, USA). Probes DNA sequences are listed in Supplementary Table 2.

### Lactate level measurement

Lactate levels in mouse hearts were measured using the Lactate Colorimetric Assay Kit II (BioVision, Milpitas, USA). Each heart was homogenated in 1 mL of ice-cold lactate assay buffer in the kit using TissueRuptor (Qiagen, Hilden, Germany). The lysate was centrifuged at 10,000 × *g* for 5 min, and the supernatant was used for lactate measurement according to the manufacturer’s protocol.

### Mitochondrial fractionation

Mitochondria were isolated from fresh mouse heart tissues essentially as previously described in refs. ^15,16^. Briefly, dissected heart tissue was cut into small pieces on ice with scissors and then homogenized in 5 mL of extraction buffer [225 mM mannitol, 75 mM sucrose, 10 mM HEPES-KOH (pH 7.6), 2 mM EDTA, Protease inhibitor cocktail (Roche), and 0.0025% 2-mercaptoethanol] using a Teflon homogenizer with 15 strokes at 700 rpm, maintaining cooling on ice. The homogenate was centrifuged at 600 × *g* for 10 min at 4 °C. Subsequently, the supernatant was transferred to a new tube and centrifuged at 7000 × *g* for 10 min to acquire the mitochondrial fraction pellet. The mitochondrial fraction pellet was resuspended in the extraction buffer and adjusted to 1 mg mL^−1^ using a protein assay kit (Bio-Rad, Hercules, USA). The mitochondrial fraction was used for subsequent blue native-PAGE and respiratory complex activity measurements.

### Blue native-PAGE

Blue native-PAGE was performed as previously described in ref. ^15^. The mitochondrial fraction containing 125 μg of protein was suspended in 40 μL of solubilizing buffer containing 50 μM bis-Tris (pH 7.0), 1 M aminocaproic acid, and 1.5 % DDM (*n*-dodecyl β-d-maltoside). Samples were cleared by centrifuging at 100,000 × *g* for 15 min at 4 °C. The supernatant was mixed with 3 μL of brilliant blue G (dissolved in 1 M aminocaproic acid). About 20 μL of the sample was subjected to blue native-PAGE using a 3–12% Bis-Tris native gel (Invitrogen, Carlsbad, USA). Once the dye traveled one-third of the gel length, the first cathode buffer was replaced with the second cathode buffer (10^−1^ dilution of the first cathode buffer).

### Western blot

Western blot was performed essentially as previously described in ref. ^44^. Tissues were homogenized in lysis buffer (150 mM NaCl,100 mM Tris-HCl pH 8, 0.5% NP-40, and protease inhibitor cocktail (Roche, Basel, Switzerland)) and sonicated for 10 s. The protein concentration was determined using a BCA protein assay kit (Thermo Fisher Scientific, Waltham, USA). Samples were electrophoresed in SDS polyacrylamide gel and transferred to an Immobilon-P membrane (Merck Millipore, Billerica, USA). The membrane was blocked with 5% skim milk in TBST buffer (150 mM NaCl, 25 mM Tris-HCl pH 7.4, 2.7 mM KCl, and 0.05% Tween-20) and probed for respective proteins using the primary antibodies diluted in 5% skim milk in TBST buffer at 4 °C, overnight. The membrane was washed in TBST and was probed using the secondary antibody at room temperature for 1 h, followed by washing in TBST. The signals were detected using ECL Prime Western Blotting Detection Reagent (GE Healthcare, Chicago, USA) and an ImageQuant 400 imager (GE Healthcare). The antibodies and their conditions for use are listed in Supplementary Table 3.

### Respiratory complex activity

The mitochondrial fraction (1 mg mL^−1^) was briefly sonicated before use and the activities of complexes I, II, III, and IV were measured essentially as previously described in refs. ^15,49^. For complex I activity measurement, 980 μL of the solution containing 50 mM potassium phosphate (pH 7.4), 2 mM KCN, 75 μM NADH (Nicotinamide adenine dinucleotide reduced disodium salt), and 50 μM Coenzyme Q1, was mixed and incubated at 30 °C for 3 min. Subsequently, 20 μL (20 μg) of mitochondrial protein was added and absorbance at 340 nm was measured for 200 s. Enzymatic activity was calculated using the extinction coefficient of NADH (6.22 mM^−1^ cm^−1^).

For complex II activity measurement, 965 μL of reaction solution containing 50 mM potassium phosphate (pH 7.4), 20 mM succinate, and 20 μg of mitochondrial protein was mixed and incubated at 30 °C for 10 min. Subsequently, final concentrations of 2 μg mL^−1^ of Antimycin A, 2 μg mL^−1^ of rotenone, 2 mM KCN, 50 μM DCPIP (2,6-Dichloroindophenol sodium salt hydrate), and DB (decylubiquinone) were added and absorbance at 600 nm was measured for 200 s. Enzymatic activity was calculated using the extinction coefficient of DCPIP (19.1 mM^−1^ cm^−1^).

Prior to complex III activity measurement, we prepared DBH_2_ solution by mixing 100 μL of DB with 10 mg of potassium borohydride and 10 μL of 100 mM HCl. The supernatant was transferred to a new tube and 5 μL of 1 M HCl was added. For complex III activity measurement, 984 μL of reaction solution containing 10 mM potassium phosphate (pH 7.4), 50 μM cytochrome C, 1 mM EDTA, 2 mM KCN, and 4 μM rotenone was mixed and incubated at 30 °C for 10 min. Subsequently, 10 μg (10 μL) of mitochondrial protein and 6 μL of DBH_2_ solution were added and absorbance at 550 nm was measured for 200 s. Enzymatic activity was calculated using the extinction coefficient of cytochrome c (19.0 mM^−1^ cm^−1^).

Prior to complex IV activity measurement, 2.7 mg of cytochrome c was dissolved in MilliQ water and 5 μL of 100 mM dithiothreitol was added and incubated for >15 min at room temperature in the dark. For complex IV activity measurement, 1 mL of reaction solution containing 10 mM potassium phosphate (pH 7.4), 50 μL of cytochrome c, and 10 μL (10 μg) of mitochondrial proteins was mixed and absorbance at 550 nm was measured for 200 s. Enzymatic activity was calculated using the extinction coefficient of cytochrome c (19.0 mM^−1^ cm^−1^).

For citrate synthase activity measurement, 1 mL of reaction solution containing 100 mM Tris-HCl (pH 8.0), 300 mM acetyl-coA, 0.1 mM DTNB (5,5′-dithiobis 2-nitrobenzoic acid), 0.5 mM oxaloacetate, and 10 μL (10 μg) of mitochondrial proteins were mixed and absorbance at 412 nm measured for 200 s. Enzymatic activity was calculated using the extinction coefficient of TNB (thionitrobenzoic acid) (13.6 mM^−1^ cm^−1^).

### Statistics and reproducibility

All numerical data were analyzed by GraphPad Prism 9 software. All the “*n*” corresponds to individual animals. Three to five animals were used for each group to confirm reproducibility and minimize animal sacrifice. No data were excluded. Control and KO animals were tested in the order of Control 1, KO1, Control 2, KO2, Control 3, KO3,… unless otherwise noted to minimize time bias in experiments. Blinding was not performed unless otherwise noted, due to constraints of time and personnel. To assess differences between the two groups, Welch’s *t*-test was used unless otherwise noted. A two-tailed *P* value of 0.05 was considered significant. To assess differences between four groups with two variables, a two-way analysis of variance (ANOVA) followed by Tukey’s test was used. Data were presented as means ± standard error of means (s.e.m.).

### Reporting summary

Further information on research design is available in the Nature Portfolio Reporting Summary linked to this article.

## Supplementary information

Supplementary Information

Description of Additional Supplementary Files

Supplementary Data

Reporting Summary

## Data Availability

The source data underlying the graphs are provided as Supplementary Data, and the uncropped and unedited gel and western blot images are provided as Supplementary Fig. 1. All data presented in this study are available upon request.
