# Supplementary material for: NSUN3-mediated mitochondrial tRNA 5-formylcytidine modification is essential for embryonic development and respiratory complexes in mice
Source: Commun Biol. 2023 Mar 22;6:307. doi: 10.1038/s42003-023-04680-x (PMC10033821; doi:10.1038/s42003-023-04680-x)
Supplement: Supplementary file 1 — Supplementary Information [file 42003_2023_4680_MOESM1_ESM.pdf]

Supplementary Information for

NSUN3-mediated mitochondrial tRNA 5-formylcytidine modification is essential for embryonic development and respiratory complexes in mice

Yoshitaka Murakami, Fan-Yan Wei, Yoshimi Kawamura, Haruki Horiguchi, Tsuyoshi Kadomatsu, Keishi Miyata, Kyoko Miura, Yuichi Oike, Yukio Ando, Mitsuharu Ueda, Kazuhito Tomizawa*, and Takeshi Chujo*

*Corresponding authors. Email:
tchujo@kumamoto-u.ac.jp (T.C.), tomikt@kumamoto-u.ac.jp (K.T.)

This file includes:

Supplementary Figures 1, 2, 3, 4 and their legends.

Supplementary Tables 1, 2 and 3.

Supplementary Figure 1. Uncropped gel and western blot images.

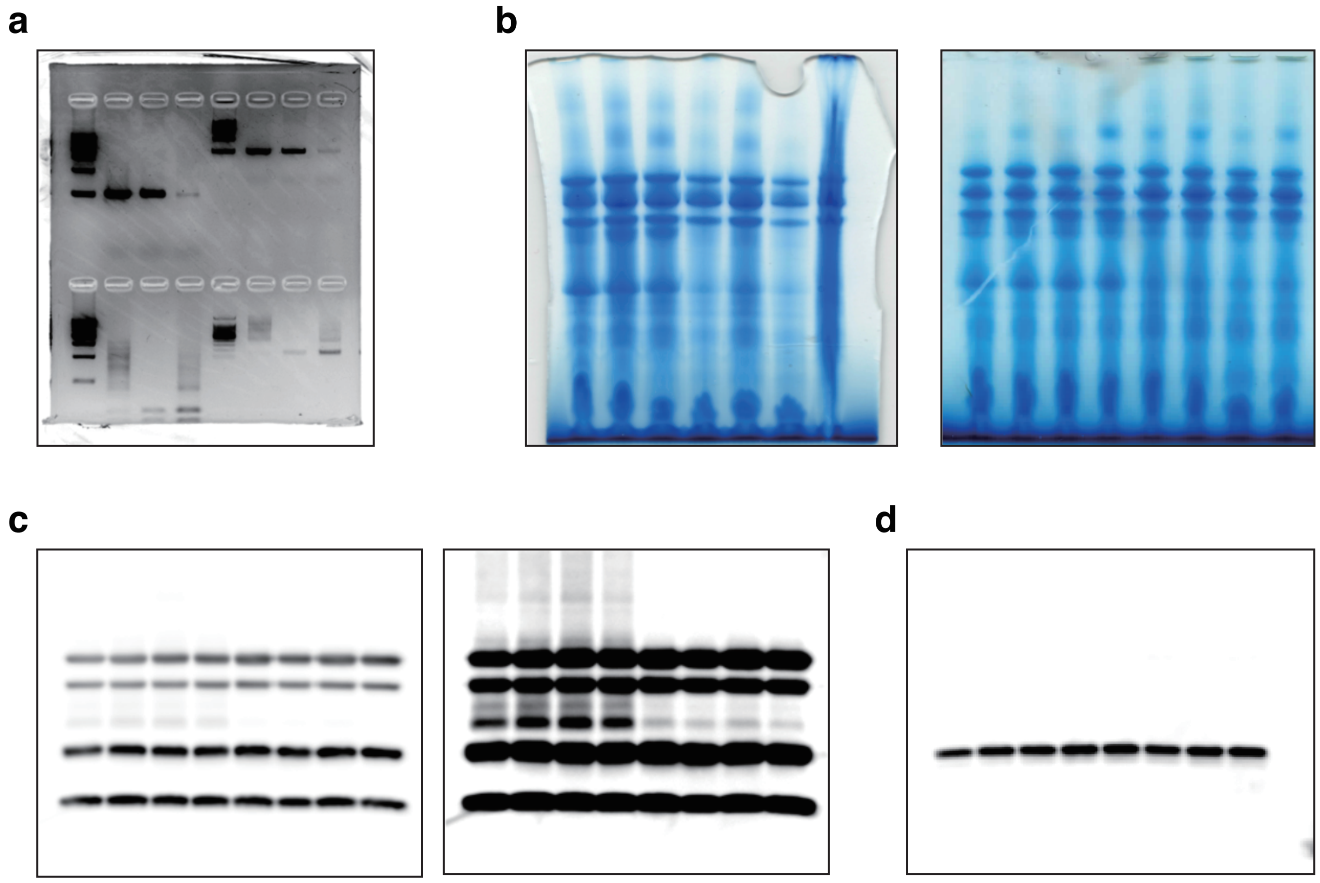

Supplementary Figure 1. Uncropped gel and western blot images.
a Uncropped agarose gel image of Figure 1d. Upper left area corresponds to the WT allele PCR products, and lower right area corresponds to the *Nsun3* KO allele PCR products. A thin band seen in WT allele PCR of *Nsun3* -/- embryo likely derives from a small amount of heterozygous mother cell contamination. **b** Uncropped blue native PAGE gel images of Figure 6a (left) and 6b (right). **c** Uncropoped western blot image of Figure 6c respiratory complex proteins. The same image of the membrane probed by Mitochondrial OXPHOS antibodies cocktail (Abcam) is shown in two different contrasts for visibility of different proteins. **d** Uncropped VDAC1 western blot image of Figure 6c.

Supplementary Figure 2. Whole membrane images of northern blots in Figure 5.

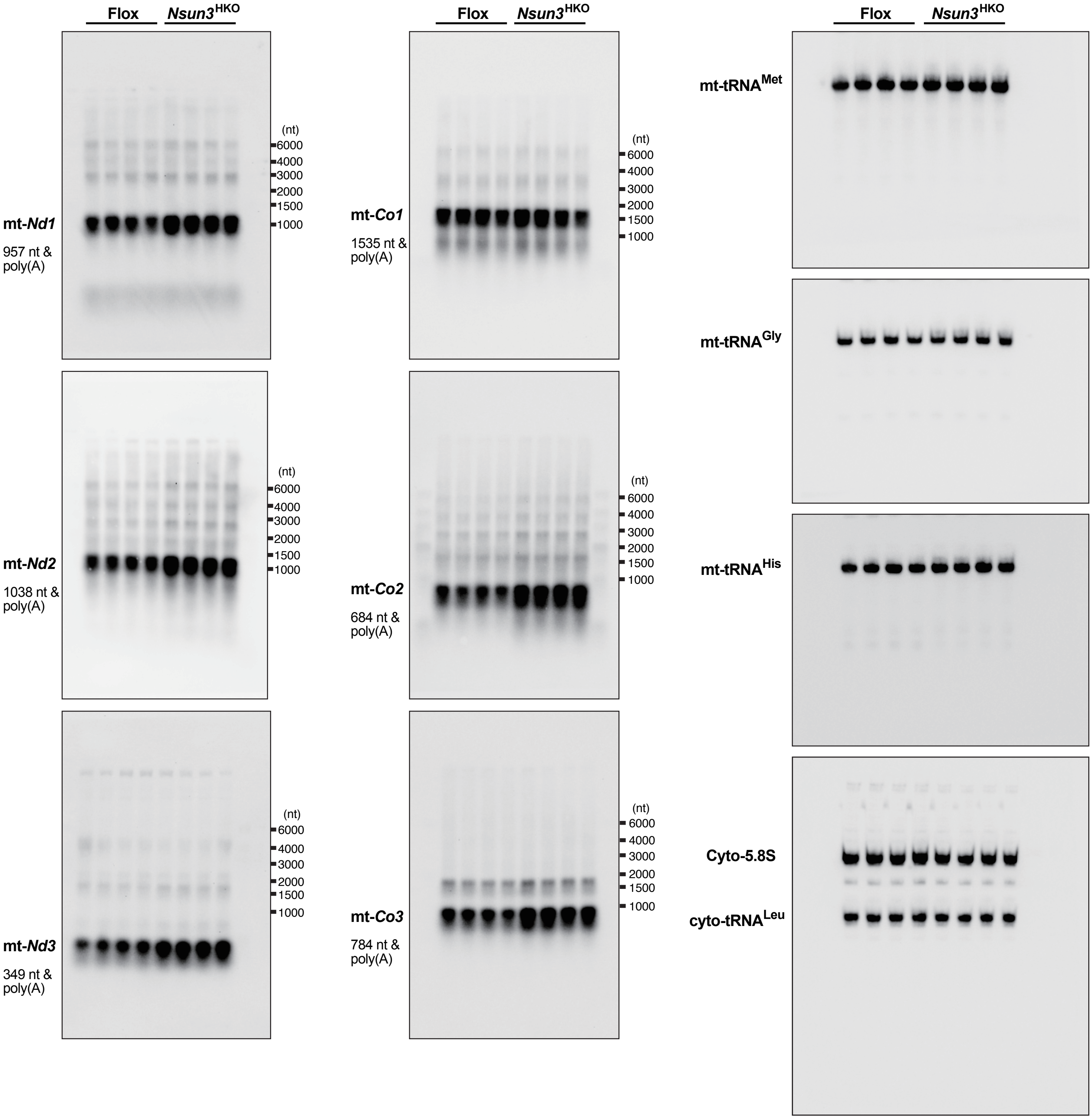

Supplementary Figure 2. Whole membrane images of northern blots in Figure 5.
On the left side of mt-mRNA northern blots, the probe names and the expected sizes of mRNAs without polyadenylation are displayed. mt-mRNAs typically have oligo(A) tails (consisting of several nucleotides) or poly(A) tails (up to around 50 nucleotides). On the right side of the mRNA northern blots, the location of RNA size markers, which were stained with methylene blue and marked on the membrane with a pencil, are illustrated.

Supplementary Figure 3. Equivalent levels of unfolded protein stress marker mRNAs in *Nsun3*^{HKO} heart.

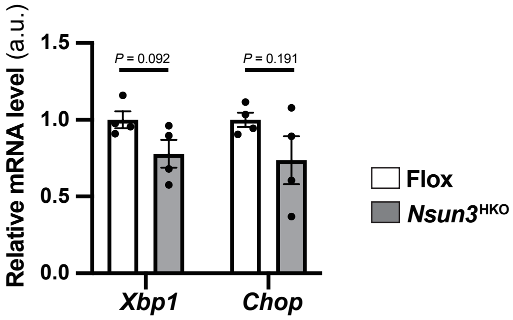

Supplementary Figure 3. Equivalent levels of unfolded protein stress marker mRNAs in *Nsun3*^{HKO} hearts. Unfolded protein response marker mRNAs *Xbp1* and *Chop* in mouse hearts were quantified by RT-qPCR and their levels were normalized by *Actb* mRNA levels. Means ± s.e.m. from n = 4 mice. a.u., arbitrary units. Welch's *t*-test was performed.

Supplementary Figure 4. Whole-body *Nsun3* KO embryos at E9.5.

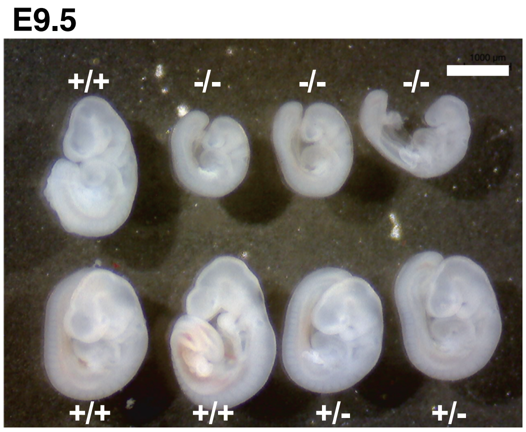

Supplementary Figure 4. Whole-body *Nsun3* KO embryos at E9.5.
The morphology of WT (+/+), heterozygous (+/-) and KO (-/-) embryos at stage E9.5 from the uterus of a heterozygous mother mouse were observed under a stereomicroscope. Scale bar, 1 mm.

Supplementary tables

Supplementary Table 1. Initiation codons of mammalian mt-mRNAs

Complex, mt-mRNA	*Homo sapiens* (Human) NC_012920	*Mus musculus* (Mouse) NC_005089	*Bos taurus* (Cow) NC_006853	*Sus sucrofa* (Pig) NC_000845	*Canis lupus* (Dog) NC_002008	*Felis catus* (Cat) NC_001700	*Loxodonta africana* (Elephant) NC_00934	*Didelphis virginiana* (Opossum) NC_001610
I, mt-*ND1*	AUA	GUG	AUG	AUG	AUG	AUG	AUG	AUA
I, mt-*ND2*	AUU	AUA	AUA	AUU	AUA	AUC	AUA	AUG
I, mt-*ND3*	AUA	AUU	AUA	AUA	AUA	AUA	AUA	AUA
I, mt-*ND4*	AUG	AUG	AUG	AUG	AUG	AUG	GUG	AUG
I, mt-*ND4L*	AUG	AUG	AUG	GUG	AUG	AUG	AUG	AUA
I, mt-*ND5*	AUA	AUC	AUA	AUA	AUA	AUA	AUA	AUA
I, mt-*ND6*	AUG	AUG	AUG	AUG	AUG	AUG	AUG	AUA
III, mt-*CYTB*	AUG	AUG	AUG	AUG	AUG	AUG	AUG	AUG
IV, mt-*CO1*	AUG	AUG	AUG	AUG	AUG	AUG	AUG	AUG
IV, mt-*CO2*	AUG	AUG	AUG	AUG	AUG	AUG	AUG	AUG
IV, mt-*CO3*	AUG	AUG	AUG	AUG	AUG	AUG	AUG	AUG
V, mt-*ATP6*	AUG	AUG	AUG	AUG	AUG	AUG	GUG	AUG
V, mt-*ATP8*	AUG	AUG	AUG	AUG	AUG	AUG	AUG	AUG

NCBI Refseq numbers of mitochondrial genomes are indicated by ‘NC_number’ below the species name. AUG, black; AUA, red; other initiating codons, blue.

Supplementary Table 2. Oligo DNAs used in this study.

**Genotyping**	
Nsun3 wild-type allele forward	TGGAAGGGGAACACACAGTG
Nsun3 wild-type allele reverse	ATGTGGTGCCTCCCCATTTT
Nsun3 KO allele forward	TGAAGATAGAGTGACTCGGGC
Nsun3 KO allele reverse	CCAAGTGCTTGAGAGGGCAT
Nsun3 Flox forward	CACTTCCTGAAGATGAGTAGCTTTT
Nsun3 Flox reverse	CTGGCCTAGAACTCAGAGAACATCT
Cre forward	ACATGTTCAGGGATCGCCAG
Cre reverse	TAACCAGTGAAACAGCATTGC
**qPCR**	
Actb forward	AGAAAATCTGGCACCACACC
Actb reverse	CAGAGGCGTACAGGGATAGC
Nsun3 Ex3-Ex4 forward	ATCCGTAGCTCTGCTGCAAT
Nsun3 Ex3-Ex4 reverse	ATCAAAGGCTGTGGGATGAA
Xbp1 forward	CACAAGGCCGTGAGTTTTCT
Xbp1 reverse	GAGCAGCAAGTGGTGGATTT
Chop forward	CGCAGGGTCAAGAGTAGTGAA
Chop reverse	GAGCTGGAAGCCTGGTATGA
**Northern blot probes**	
mt-tRNA^{Met}	GTATGGGCCCGATAGCTTAATTAGCTGACCTTACT
mt-tRNA^{Gly}	TCTGGGTTTATTCAGAATCTACTAATTGGAAGTCAG
mt-tRNA^{His}	AAGGAGGTTTATTTCCTGTTGTCAGATTCACAGTC
cyto-tRNA$^{Leu}_{CAA}$	TGTCAGAAGTGGGATTCGAACCCACGCCTC
cyto-5.8S rRNA	GCAAGTGCGTTCGAAGTGTCGATGATCAAT
mt-*Nd1* mRNA	ATATGAAATTGTTTGGGCTACGGCTCGTAA
mt-*Nd2* mRNA	TGAGGTTGAGTAGAGTGAGGGATGGGTTGT
mt-*Nd3* mRNA	GTTTGAATTGCTCATGGTAGTGGAAGTAGA
mt-*Co1* mRNA	AATATTACCTCCGTGTAGGGTTGCAAGTCA
mt-*Co2* mRNA	CCAGGTTTTAGGTCGTTTGTTGGGATTATA
mt-*Co3* mRNA	AGTAGTGGGACTTCTAGAGGGTTAAGTGGT

Supplementary Table 3. Antibodies used in this study.

Antibody	Animal, Producer, Catalog number, Dilution
Mitochondrial OXPHOS (rodent)	Mouse, Abcam, ab110413, 1:5000
VDAC1	Mouse, Abcam, ab14734, 1:2000
Anti-Mouse HRP	Goat, Dako, P0447, 1:2000
