# Supplementary material for: NSUN3-mediated mitochondrial tRNA 5-formylcytidine modification is essential for embryonic development and respiratory complexes in mice
Source: Commun Biol. 2023 Mar 22;6:307. doi: 10.1038/s42003-023-04680-x (PMC10033821; doi:10.1038/s42003-023-04680-x)
Supplement: Supplementary file 4 — Reporting Summary [file 42003_2023_4680_MOESM4_ESM.pdf]

nature portfolio

Corresponding author(s): Takeshi Chujo; Kazuhito Tomizawa

Last updated by author(s): Feb 20, 2023

Reporting Summary

Nature Portfolio wishes to improve the reproducibility of the work that we publish. This form provides structure for consistency and transparency in reporting. For further information on Nature Portfolio policies, see our Editorial Policies and the Editorial Policy Checklist.

Statistics

For all statistical analyses, confirm that the following items are present in the figure legend, table legend, main text, or Methods section.

n/a	Confirmed	
☐	☒	The exact sample size (*n*) for each experimental group/condition, given as a discrete number and unit of measurement
☐	☒	A statement on whether measurements were taken from distinct samples or whether the same sample was measured repeatedly
☐	☒	The statistical test(s) used AND whether they are one- or two-sided<br>*Only common tests should be described solely by name; describe more complex techniques in the Methods section.*
☒	☐	A description of all covariates tested
☐	☒	A description of any assumptions or corrections, such as tests of normality and adjustment for multiple comparisons
☐	☒	A full description of the statistical parameters including central tendency (e.g. means) or other basic estimates (e.g. regression coefficient) AND variation (e.g. standard deviation) or associated estimates of uncertainty (e.g. confidence intervals)
☐	☒	For null hypothesis testing, the test statistic (e.g. *F*, *t*, *r*) with confidence intervals, effect sizes, degrees of freedom and *P* value noted<br>*Give P values as exact values whenever suitable.*
☒	☐	For Bayesian analysis, information on the choice of priors and Markov chain Monte Carlo settings
☒	☐	For hierarchical and complex designs, identification of the appropriate level for tests and full reporting of outcomes
☒	☐	Estimates of effect sizes (e.g. Cohen's *d*, Pearson's *r*), indicating how they were calculated

Our web collection on statistics for biologists contains articles on many of the points above.

Software and code

Policy information about availability of computer code

Data collection	Codes were not used.
Data analysis	Codes were not used.

For manuscripts utilizing custom algorithms or software that are central to the research but not yet described in published literature, software must be made available to editors and reviewers. We strongly encourage code deposition in a community repository (e.g. GitHub). See the Nature Portfolio guidelines for submitting code & software for further information.

Data

Policy information about availability of data

All manuscripts must include a data availability statement. This statement should provide the following information, where applicable:

- Accession codes, unique identifiers, or web links for publicly available datasets
- A description of any restrictions on data availability
- For clinical datasets or third party data, please ensure that the statement adheres to our policy

All data presented in this study are available upon request.

Human research participants

Policy information about studies involving human research participants and Sex and Gender in Research.

Reporting on sex and gender	Not a human research.
Population characteristics	Not a human research.
Recruitment	Not a human research.
Ethics oversight	Not a human research.

Note that full information on the approval of the study protocol must also be provided in the manuscript.

Field-specific reporting

Please select the one below that is the best fit for your research. If you are not sure, read the appropriate sections before making your selection.

☒ Life sciences ☐ Behavioural & social sciences ☐ Ecological, evolutionary & environmental sciences

For a reference copy of the document with all sections, see nature.com/documents/nr-reporting-summary-flat.pdf

Life sciences study design

All studies must disclose on these points even when the disclosure is negative.

Sample size	Three to five animals were used for each group, to confirm reproducibility and minimize animal sacrifice.
Data exclusions	No data were excluded.
Replication	All the ‘n’ corresponds to individual animals, and reproducibility was confirmed between individuals.
Randomization	Control and KO animals were tested in the order of Control 1, KO1, Control 2, KO2, Control 3, KO3, ... unless otherwise noted, in order to minimize time bias in experiments.
Blinding	Blinding was not performed unless otherwise noted, due to constraints of time and personnel.

Reporting for specific materials, systems and methods

We require information from authors about some types of materials, experimental systems and methods used in many studies. Here, indicate whether each material, system or method listed is relevant to your study. If you are not sure if a list item applies to your research, read the appropriate section before selecting a response.

Materials & experimental systems

n/a	Involved in the study	
☐	☒	Antibodies
☒	☐	Eukaryotic cell lines
☒	☐	Palaeontology and archaeology
☐	☒	Animals and other organisms
☒	☐	Clinical data
☒	☐	Dual use research of concern

Methods

n/a	Involved in the study	
☒	☐	ChIP-seq
☒	☐	Flow cytometry
☒	☐	MRI-based neuroimaging

Antibodies

Antibodies used	Mitochondrial OXPHOS (rodent), Abcam, ab110413; VDAC1, Abcam, ab14734;<br>Anti-Mouse HRP, Dako, P0447
Validation	The band sizes were monitored and compared to the manufacturer's data sheet and/or previous research papers.

March 2021

Animals and other research organisms

Policy information about studies involving animals; ARRIVE guidelines recommended for reporting animal research, and Sex and Gender in Research

Laboratory animals	C57BL/6J mice were used, and samples were collected from the mice at 14 or 50 weeks of age.
Wild animals	The study did not involve wild animals.
Reporting on sex	Male mice were utilized for experiments, while female mice were primarily utilized for breeding purposes.
Field-collected samples	The study did not involve samples collected from the field.
Ethics oversight	All animal procedures were approved by the Animal Ethics Committee of Kumamoto University.

Note that full information on the approval of the study protocol must also be provided in the manuscript.
